# Interpretable machine learning analysis to identify risk factors for diabetes using the anonymous living census data of Japan

**DOI:** 10.1007/s12553-023-00730-w

**Published:** 2023-01-26

**Authors:** Pei Jiang, Hiroyuki Suzuki, Takashi Obi

**Affiliations:** 1 Course of Information and Communication, Department of Engineer, Tokyo Institute of Technology, Kanagawa, Japan; 2 Present Address: 4259 Nagatsutachou, Midori Ward, Yokohama, Kanagawa, 226-0026 Japan; 3 Center for Mathematics and Data Science, Gunma University, Maebashi, Gunma Japan; 4 Institute of Innovative Research, Tokyo Institute of Technology, Kanagawa, Japan

**Keywords:** Interpretable machine learning, Non-objective-oriented census data, Diabetes, Risk factors

## Abstract

**Purpose:**

Diabetes mellitus causes various problems in our life. With the big data boom in our society, some risk factors for Diabetes must still exist. To identify new risk factors for diabetes in the big data society and explore further efficient use of big data, the non-objective-oriented census data about the Japanese Citizen’s Survey of Living Conditions were analyzed using interpretable machine learning methods.

**Methods:**

Seven interpretable machine learning methods were used to analysis Japan citizens’ census data. Firstly, logistic analysis was used to analyze the risk factors of diabetes from 19 selected initial elements. Then, the linear analysis, linear discriminate analysis, Hayashi’s quantification analysis method 2, random forest, XGBoost, and SHAP methods were used to re-check and find the different factor contributions. Finally, the relationship among the factors was analyzed to understand the relationship among factors.

**Results:**

Four new risk factors: the number of family members, insurance type, public pension type, and health awareness level, were found as risk factors for diabetes mellitus for the first time, while another 11 risk factors were reconfirmed in this analysis. Especially the insurance type factor and health awareness level factor make more contributions to diabetes than factors: hypertension, hyperlipidemia, and stress in some interpretable models. We also found that work years were identified as a risk factor for diabetes because it has a high coefficient with the risk factor of age.

**Conclusions:**

New risk factors for diabetes mellitus were identified based on Japan's non-objective-oriented anonymous census data using interpretable machine learning models. The newly identified risk factors inspire new possible policies for preventing diabetes. Moreover, our analysis certifies that big data can help us find helpful knowledge in today's prosperous society. Our study also paves the way for identifying more risk factors and promoting the efficiency of using big data.

## Introduction

Diabetes Mellitus (DM) not only influences our daily life but also causes various complications, such as Ketoacidosis, hypertension, kidney disease, foot complications, etc. [1]. World Health Organization (WHO) reports that 422 million adults have DM around the world, which makes one of every eleven people a DM patient [2]. In Japan, the prevalence of diabetes has been steadily increasing and is expected to grow by 10% in 2030 [3]. Moreover, researchers found that diabetes patients are easier to have COVID-19 [4]. To prevent the severe effects caused by DM, many institutions make various efforts to prevent diabetes. WHO publishes a yearly report regarding diabetes [2]. The US Centre for Disease Control and Prevention initiated the National Diabetes Prevention Program to prevent or delay type 2 diabetes [5]. Certification Board for Diabetes Educators in Japan [6] trains doctors and nurses with the essentials to assisting diabetes patients. Japan Preventive Association of Life-style Related Disease [7] is trying to inform citizens how to prevent diabetes by improving good life habits. The Japan Diabetes Society [8] organizes an annual conference and promotes diabetes research. Diabetes Network [9] of Japan collects data about diabetes and organizes various events to educate the public and help prevent diabetes in Japan.

Even though governments have made various efforts to prevent diabetes mellitus, the profound influence caused by diabetes still exists. And because of COVID -19, our life habits changed. Efforts to stop DM are still necessary. Identifying new risk factors of DM can help us make more efficient policies to prevent DM. Therefore, researchers made various efforts to find new associated risk factors for DM. For example, Aidin et al. [10] found a relationship between diabetes patients’ mortality and cardiovascular disease. Meanwhile, age [11] and gender [11, 12] were also identified as affecting the prevalence of DM. At the same time, a dietary factor of diabetes was found in some studies [13–15]. Moreover, several metabolic and anthropometric traits were associated with DM: BMI [16], overweight [17–19] and obesity [17, 18, 20] were found as associated factors for DM. Considering lifestyle and environmental factors, more risk factors associated with DM were identified: social-economic statics [21], life environment [22], life habits [23] and lifestyle [24] smoking status [25–38] or cigarette consumption [10, 13, 26], alcohol consumption [13, 39], occupation [40], work stress [12, 41], work years [40, 42], weekly work hours [41]. Research by Bellou et al. [13] indicated that a low level of education and conscientiousness decreased physical activity, high sedentary time, duration of television watching, and air pollution presented robust evidence for increased risk of type 2 DM. Although current works have achieved great success, all their factors were identified by objective-oriented datasets. They ignore that we are in a prosperous data society with the technological development of the Internet of Things (IoT). Furthermore, these methods cannot have the ability to deal with large-scale complex DM-related risk factors analysis in the current big data society. Thus, there is a crude need to design a model to identify more risk factors of large-scale complex DM-related data.

Fortunately, with the development of technology, various reliable and robust machine learning methods [43–45] were proposed and used to classify or predict complex risk factors of multiple diseases. Significantly, the Interpretable Machine Learning (IML) models will capture the “extraction of relevant knowledge from a machine-learning model concerning relationships either contained in data or learned by the model” [46]. Especially with the development of AI technology, the explainable AI (XAI) models help us understand the black box AI model and provide a local or global explanation of models. The robust XAI or explainable machine learning ( XML) methods LIME [47] and SHAP [48] methods were used in various fields and were certificated efficient, especially in medical and clinical areas. Some research confirmed that LIME [49–52] and SHAP [51–55] could be used to explain models and give reasons for model decisions. Because of the robustness of XML methods, we used them to analyse our data and acquired perfect/hoped results.

Therefore, the IML methods were used to analyse the non-objective-oriented data helping us identify new risk factors for DM in our study. The knowledge from IML models also will help us take better actions to prevent DM for private persons and guide governments’ policymaking. Therefore, we started this study hoping to find new knowledge about DM using Japan's non-objective-oriented anonymous census data. The significant contributions of our study are as follows:

• We proposed using interpretable machine learning to obtain new risk factors that suggest DM prevention in current society.
• As far as we know, our analysis is a significant new try that uses non-objective-oriented data to find knowledge in the booming big data society.
• Our study paved the way for finding more useful knowledge using interpretable machine learning methods.

The rest of the paper is organized as follows. Section 2 describes the used datasets. The methodology in this analysis is introduced in Section 3. Section 4 shows the details results of our study. Section 5 shows the discussion of our results and the limitations of our study. Finally, Sect. 6 discusses the future direction and concludes the paper.

## Data source

Generally, research analysis for DM is based on experimental-designed statistical data. However, in our current society, there are various kinds of data. Using the non-objective oriented data to find knowledge is necessary. Therefore, we analyzed the Japanese citizens living survey data, which were collected to know the daily life of Japanese citizens by the Ministry of Healthcare, Labor, and Welfare (MHLW) [56] in Japan, hoping to find new risk factors for DM. MHLW compiles a comprehensive census of Japanese citizens living every four years since 1995, which includes many information items: personal information (age, sex, marriage situation, etc.); information about work (profession, company description, weekly work hours, weekly workdays, and work years since starting work); information about the family (number of family members, etc.); living situation (the space of houses, the numbers of rooms, etc.); hospital information and healthcare situation (stress, various diseases information, etc.). These anonymous census data can help the Japanese government understand the citizens’ living conditions and promote citizen living. Because the MHLW data collection process is not designed specifically for DM, the census data without object-oriented design makes identifying the hidden risk factors for DM possible. The census data from 2013 was used in this analysis. And 28,292 samples were extracted from 192,519 anonymous samples by deleting the samples with missed values. The extracted samples contain 19 factors: number of family members, spending of one year (Spence), room space, number of rooms for one family (room_num), age, sex, insurance type in Japan, national public pension type in Japan, weekly work hours, weekly workdays, total work years, profession category, obesity, hyperlipidemia, hypertension, healthcare awareness level, health investigation situation in Japan, stress, and smoking status. Among all the factors, sex, obesity, hyperlipidemia, hypertension, and anxiety are dichotomous, whose values are defined as binarized value: 0 or 1, while other factors’ value is ordinal. All the samples were analyzed using seven interpretable machine-learning methods.

## Methodology

With the development of analysis technology, the reliability of models is also essential, besides model accuracy. The interpretable machine learning models can make decisions and tell us why one decision was made. Therefore, we used interpretable machine learning models to analyze the MHLW census data. Generally interpretable machine learning (IML) models have two kinds of types: intrinsic (rule-based) or post hoc models [57]. The intrinsic models obtain knowledge by restricting the rules of machine learning models. In contrast, the post hoc models refer to the application of interpretation after training, such as Local interpretable model-agnostic explanations (LIME) [47] and Shapley Additive explanations (SHAP [48]. Primarily, the SHAP method was used in various filed and was certificated robust [58–63]. Therefore, we used SHAP to explain the multi-layer perception (MLP) model to calculate the feature importance. The intrinsic models generally contain Logistic Analysis (LA), linear regression, Linear discrimination (LDA), Hayashi’s quantification method 2 (qt2) [64], random forest, and XGBoost methods. In our study, we used both models to find and check the feature contributions in the DM classification.

**Fig. 1:**
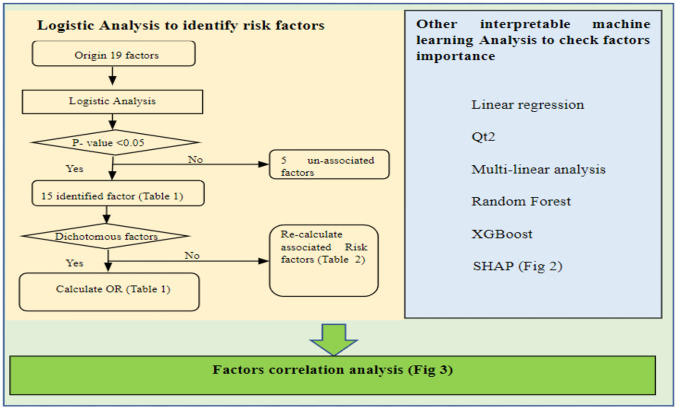
Flow chart of risk factor analysis

Firstly, the commonly used logistic analysis was used to find the associated risk factors of DM. Other IML methods were used to check the importance of each factor. Logistic analysis is one of the most widely used methods to analyze statistical data in biology and healthcare fields (Table 4). Using logistic analysis, we will not only be able to identify related factors like linear regression but also can use it to predict the possibility of disease occurrence. Meanwhile, LA can also check the risk increase for factors by calculating the odds ratio (OR), especially for dichotomous factors (Table 1). The detailed steps of our analysis are shown in Fig. 1. Firstly, all aspects were tested using a univariate logistic regression model, and 15 strongly related factors (P < 0.05) were identified. Then the OR of all dichotomous variables was calculated (Table 2). The associated risk factors of DM were rechecked using multiple linear regression to review multi-related risk factors of DM (Table 3). Consequently, associated risk factors of DM were identified (Table 1). The OR of associated risk factors for ordinal factors was also checked (Table 2).

**Table 1:** Identified risk factors of DM using logistic analysis

Items		Data volume	P-value	The odds ratio of Diabetes
**Age**	15 ~	28,923	< 0.001	Table 2
**Sex**	Male (values in data: 1)	15,930	< 0.001	2.7928
	Female (values in data: 0)	12,992		
**Number of family members**	Seven categories	28,923	0.0037	Table 2
**Obesity**	Have (values in data: 1)	433	< 0.001	6.0977
	no (values in data: 0)	28,490		
**Hyperlipidemia**	have (values in data: 1)	3281	< 0.001	1.3713
	no (values in data: 0)	25,642		
**Hypertension**	have (values in data: 1)	7171	< 0.001	1.2597
	no (values in data: 0)	21,752		
**Insurance type**	Five types	28,923	0.0030	Table 2
**Public pension**	Four categories	28,923	< 0.001	Table 2
**Health awareness level**	Five levels	28,923	< 0.001	Table 2
**Profession**	12 kinds (S1 Table)	28,923	0.0005	Table 2
**Work time(years)**	0 ~ 50 years	28,923	0.0169	Table 2
**Week work hours**	0 ~ 80 h	28,923	0.0361	Table 2
**Week workdays**	0 ~ 7 days	28,923	0.0069	Table 2
**Stress**	yes (values in data: 1)	16,579	0.0020	1.0352
	no (values in data: 0)	10,157		
	unknown	2187	-	-
**Smoking status**	Four situations	28,923	0.0324	Table 2

Remarks: “-” stands for no calculation

**Table 2:** Associated factors of diabetes for some factors

**Factors**		**Population**	**Odds Ratio of connected Factors**														
			**Age**	**Sex**	**Family number**	**Obesity**	**Hyperlipid emia**	**Hypertension**	**Insurance type**	**National pension**	**Health awareness level**	**Profession**	**Weekly work hours**	**Weekly workdays**	**Work years**	**Stress**	**Smoking status**
Profession	1	4641	1.3	1.295	-	4.09	-	-	-	0.83	-	-	-	-	-	-	-
	2	4282	1.28	2.83	-	9.14	1.37	0.72	-	-	-	-	-	-	-	-	-
	3	3806	1.44	3.55	1.13	9.96	-	-	-	-	-	-	-	-	-	-	-
	4	2428	1.28	2.68	-	5.33	-	-	-	-	1.11	-	-	-	-	-	-
	5	4301	1.2	2.75	-	7.04	1.34	0.77	-	-	-	-	-	-	-	-	-
	6	367	1.23	-	-	4.96	-	-	-	-	-	-	-	-	-	-	-
	7	1967	1.81	-	-	8.03	-	-	-	-	-	-	-	-	-	-	-
	8	1576	1.18	3.14	-	6.33	-	-	-	-	-	-	-	-	-	-	-
	9	1973	1.27	2.81	-	9.73	-	-	-	-	1.09	-	-	-	-	-	-
	10	1022	1.26	2.33	-	3.22	-	-	-	-	-	-	-	-	-	-	-
	11	517	1.21	4.52	-	8.71	2.24		-	-	-	-	-	-	-	-	-
	12	663	1.16	4.18	-	6.75	-	-	-	-	-	-	-	-	-	-	-
	unkown	1380	-														-
Workdays	0	490	1.15	3.36	0.68	50.48	-	-	-	-	-	-	0.99	-	-	-	-
	1	384	1.19	3.28	1.36	-	-	-	-	-	-	-	-	-	-	-	-
	2	879	1.19	1.78	-	-	-	-	-	-	-	-	-	-	-	-	-
	3	1703	1.24	2.26	-	8.182	-	-	-	-	1.08	-	-	-	-	-	-
	4	2253	1.92	2.95	-	7.095	1.92	-	-	-	-	-	-	-	-	-	-
	5	13,222	1.33	2.86	-	6.252	1.33	0.77	-	0.83	1.07	1.004				0.94	-
	6	6941	1.31	2.82	-	5.967	-	0.78	-	0.87	-	-	-	-	0.99	-	-
	7	2107	1.09	2.49	-	5.363	1.49	-	-	-	1.07	-	-	-	-	-	-
	9	944	-														
Smoking status	1	18,781	1.27	2.4	-	7.45	1.29	0.84	0.93	0.88	1.06	-	-	-	-	-	-
	2	6395	1.29	3.49	-	6.04	1.32	0.76	-	0.9	1.07	1					
	3	421	-	-	-	-	-	-	-	-	-	-	-	-	-	-	-
	4	1118	1.2	-	-	-	2.7	-	0.82	-	-	-	-	-	-	1.29	-
	Unknown	2208	-														
Gender	Male	15,930	1.26	-	-	5.06	1.39	0.71	0.96	0.85	0.97	1	-	-	-	0.97	-
	Female	12,992	1.28	-	-	8.9	1.31	-	0.93	1.03	1.07	-	-	-	1	-	0.95
Family number	1	2743	1.19	2.93	-	7.618	-	-	-	-	-	-	-	-	-	-	-
	2	7903	1.22	2.82	-	5.006	1.31	0.71	-	0.92	1.08	1.004	-	-	-	0.94	-
	3	7884	1.25	2.53	-	7.09	-	-	-	0.89	-	-	-	-	-	-	-
	4	5794	1.37	3.17	-	6.877	1.66	0.74	-	0.85	1.09	-	-	-	-	0.94	-
	5	2697	1.25	3.25	-	8.508	1.87	0.68	-	-	-	-		1.18			
	6	1327	1.2	1.77	-	3.114	-	-	-	-	-	-	-	-	-	-	-
	7	575	-	3.02	-	18.61	-	-	-	-	-	-	-	-	-	-	-
Insurance type	1	8601	1.17	2.51	-	5.87	1.26	0.72	-	-	1.04	1	-	-	-	-	0.97
	2	696	1.22	4.13	-	-	2.19	-	-	-	-	-	-	0.839	-	-	-
	3	15,791	1.33	3.01	-	5.79	1.31	0.84	-	-	1.08	-	-	1.085	-	0.95	-
	4	2553	1.24	2.53	-	11.38	-	-	-	-	-	-	-	-	-	-	-
	5	953	-	3.1	-	11.99	1.74	-	-	-	-	-	-	-	-	-	-
	6	134	1.37	6.11	-	-	10.05	-	-	-	-	1.02	-	-	-	-	-
	9	unknown	-														
National pension	1	4314	1.4	3.58	-	7.65	-	0.65	-	-	-	-	-	-	-	-	-
	2	15,509	1.32	3.04	-	6.26	1.32	0.84	-	-	1.07	-	1.06	-	-	0.95	-
	3	1844	1.57	5.95	-	33.83	-	-	-	-	-	-	-	-	-	-	-
	4	7219	1.07	2.25	-	4.86	1.42	0.72	-	-	-	1	-	-	-	-	-
	unkown	37															
Health awareness level	good	2340	1.39	2.27	-	10.07	-	0.61	-	0.72	-	-	-	-	-	-	-
	Little good	4112	1.27	4.15	-	6.09	-	0.55	-	-	-	1.01	-	-	-	-	-
	general	13,883	1.23	2.76	-	5.66	1.21	0.78	0.94	0.93	-	-	-	-	-	-	-
	Little bad	5093	1.26	2.81	-	8.8	1.77	1.26	-	0.87	-	-	-	-	-	-	-
	bad	614	1.19	2.32	-	-	4.08	-	-	-	-	-	-	-	-	-	-
	unkown	2881	-														

Profession: 1: Managerial worker, 2: Professional and technical workers, 3: Clerical worker, 4: Salesperson, 5: Service workers, 6: Security worker, 7: Agriculture, forestry and fishery workers, 8: Production process workers, 9: Transport and machine operators, 10: Construction and mining workers, 11: Workers carrying, cleaning, packaging, etc. 12: Un-separated profession, 99: Unknown profession. Smoking status: 1: No smoking, 2: Smoking every day, 3: Smoking somedays, 4: Smoking over one month before, now no smoking, 9: Unknown status

**Table 3:** Risk factors rechecked by other methods

**Factors**	LDA factor contributions	qt2 factor importance	Singular Linear regression		Decision tree	
			Coefficients	P-values	Random Forest	Random Forest
**Smoking situation**	0.087	0.61	0.015	0	0.023574	0.023574
**Health investigation**	-0.1751	0.15	-0.004	0.357	0.017536	0.017536
**Health awareness level**	0.2463	1.01	0.02	0	0.044322	0.044322
**Stress**	-0.0909	0.07	-0.018	0	0.022664	0.022664
**Hypertension**	-0.2017	0.22	0.035	0	0.01921	0.01921
**Obesity**	4.6975	4.33	0.373	0	0.023565	0.023565
**Hyperlipidemia**	0.3642	0.29	0.07	0	0.014946	0.014946
**Profession**	-0.0196	0.74	0.001	0.125	0.072057	0.072057
**Weekly workdays**	-0.0038	0.34	0.003	0.036	0.044637	0.044637
**Weekly work hours**	0.0008	−	0	0.031	0.098248	0.098248
**worktime**	-0.0008	−	0.002	0	0.109439	0.109439
**Public pension**	-0.0613	0.31	0.017	0	0.021356	0.021356
**Insurance type**	-0.0183	0.6	-0.011	0	0.025507	0.025507
**Gender**	0.9447	0.83	0.082	0	0.019296	0.019296
**Age**	0.2171	2.95	0.017	0	0.07213	0.07213
**Room numbers**	0.0244	0.4	0.007	0	0.066668	0.066668
**Room space**	0	−	0	0	0.136625	0.136625
**Family numbers**	0.0148	0.33	-0.001	0.304	0.057296	0.057296
**Spence**	0.0011	−	0	0.163	0.110923	0.110923

Remarks: “-” stands for not associated significant factors

**Fig. 2:**
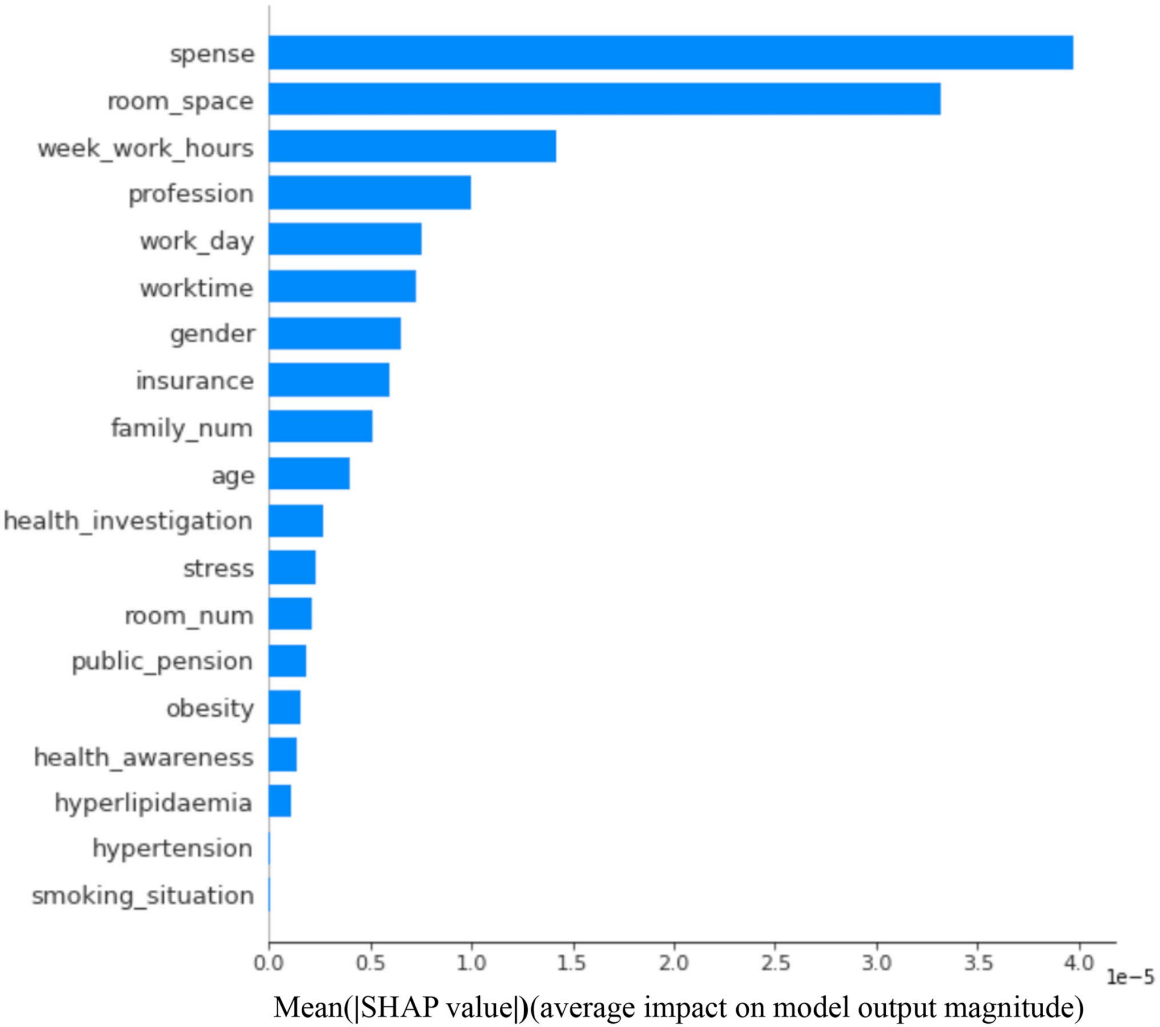
The factors of importance in SHAP analysis

**Fig. 3:**
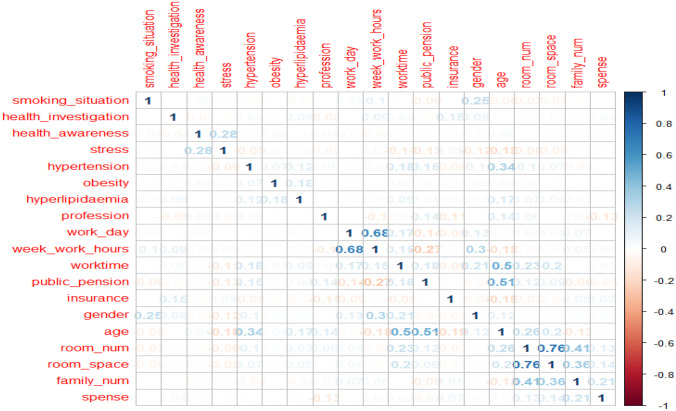
The relationship among factors

Then, the other six interpretable machine learning methods: linear regression, Linear discrimination (LDA), qt2, random forest, XGBoost, and SHAP methods, were used to recheck the factors’ importance (Table 3 and Fig. 2). In Table 3, the higher value of LDA factor contributions indicates the more critical factors in LDA analysis. In contrast, the higher qt2 factor importance means the characteristics are more acute in the qt2 elements. Similarly, the higher values in the random forest, XGBoost, and SHAP methods suggest that one factor contributes more to the classification of DM. Moreover, the co-efficient of all the factors was also checked (Fig. 3) to understand the relationship among factors. Finally, identified factors in previous research also were reviewed and compared with our analysis (Table 4).

**Table 4:** The comparison of our identified factors with other research

	**Risk factor**	**Reference**	Remarks	**Methodology**	Data type	Data region level
**Identified factors in this analysis**	Public pension	This analysis		LA &XML	NOD	National
	Health awareness	This analysis		LA &IML	NOD	National
	Insurance type	This analysis		LA &IML	NOD	National
	Family Members	This analysis		LA &IML	NOD	National
	weekly workdays	This analysis		LA & IML	NOD	National
**Factors also identified in previous research**	age	[11]		LA	OD	National
	sex	[11, 12]		LA	OD	National
	Obesity/ overweight/ BMI	[10, 16–20, 22]		A meta-analysis (MA), LA	OD	National
	hyperlipidemia	[11]		Cohort Study (CS)	OD	National
	hypertension	[11]		CS	OD	National
	smoking status	[25–28, 32–38]		LA, CS, MA, Cor proportional-hazards Regression model (CPHM), LA, CS, CPHM, multivariable-adjusted Cox regression models, CPHM, CPHM, CPHM, CPMH, CPHM, CPHM, LA	OD	National
	years of working	[40, 42]		MA, LA	OD	National
	weekly work hours	[41]		Meta-Analysis (MA)	OD	National
	profession	[40]		CS	OD	National
	stress	[12, 41],		LA	OD	National
**Factors identified by other research**	alcohol consumption	[13, 39]	$\times$	MA, LA	OD	National
	unhealthy dietary pattern	[13, 20]	$\times$	LA, UR	OD	National
	low level of education and conscientiousness	[13]	$\times$	MA	OD	National
	decreased physical activity	[13]	$\times$	MA	OD	National
	high sedentary time and duration of television watching	[13]	$\times$	MA	OD	National
	air pollution	[13]	$\times$	MA	OD	National
	some medical conditions	[13]	$\times$	MA	OD	National
	Adiposity	[13]	$\times$	MA	OD	National
	low hip circumference	[13]	$\times$	MA	OD	National
	serum biomarkers	[13]	$\times$	MA	OD	National
	Social, economic status	[21]	$\times$	Bayesian estimation	OD	National
	dietary factor	[13–15]	$\times$		OD	National
	Life environment/ habits	[22–24]	$\times$	LA, MA, LA	OD	National and City

MA method is one kind of paper review study. Therefore, it does not offer data information in paper. *NOD* Non-objective-designed, *OD* Objective-Designed. Remarks: $\times$ stands for no information item in our dataset

## Results

### The identified risk factors

After applying LA, 15 risk factors associated with DM (p < 0.005) were identified (Table 1) among the total 19 factors. Notably, insurance type, public pension, the number of family members (family_num), and health awareness level are identified as risk factors for DM using non-objective-oriented data for the first time. Meanwhile, 11 factors: age, sex, obesity, hyperlipidemia, hypertension, profession, years of working, weekly workdays, weekly work hours, stress, and smoking status were also re-identified as risk factors.

The newly identified risk factor: health awareness level was confirmed as a risk factor of DM in LA, linear analysis, qt2 analysis, and LDA analysis. The importance of health awareness level in qt2 research is 1.01, flowing the factors obesity (4.33) and age (2.95). Meanwhile, the health awareness level has comparatively higher LDA factors contribution than previous research identified: smoking situation, stress, work time, and age (Table 3). similarly, in the decision tree models, health awareness levels have higher importance than generally identified factors: stress, obesity, and gender. Data in Fig. 3 shows that health awareness level correlates more with factor stress than other factors.

Similar to the health awareness level, the public pension type in Japan was not only reconfirmed as a potential risk factor by the singular linear regression method and multiple linear regression, but it also has a higher factor contribution (0.31) than stress (0.07) and hypertension (0.22) in LDA analysis. In multiple linear regression, it wasn’t reconfirmed as a risk factor for factor insurance type. Moreover, factor insurance type’s qt2 importance (0.6) is higher than general risk factors: stress (0.07), hyperlipidemia (0.29), and hypertension (0.22). As a newly identified risk factor, family members were identified as risk factors in LA and qt2 analysis, even though family members’ factor importance of qt2 is higher than factors: stress, hypertension, and hyperlipidemia.

For the reconfirmed factors in this analysis, data from Table 1 and Table 2 show that obesity is a significant risk factor for DM, with OR over 6 (Table 1), while both the factor contribution in LDA and qt2 factor importance are over 4 (Table 3). For the commonly admitted factors: stress and smoking situation, their LDA factor contribution and qt2 factor importance are lower than the newly identified risk factor: health awareness level. Especially the factor stress, its qt2 factor importance is the weakest in the potentially associated risk factors of DM. For factor worktime (years of working), it was reconfirmed as a risk factor of DM in this analysis, while data of Fig. 3 certificates that factor worktime has a deep connection with age.

However, in the decision tree and SHAP analysis, the factor room space contributes the most to the DM classification models. In contrast, the commonly recognized risk factors of age and gender make comparatively lower contributions in XGBoost and SHAP models.

### The comparison between our analysis and other factors identification analysis

After our risk factors analysis, we compared our analysis with other studies. Our analysis creatively used various XML(Table 4) methods to identify risk factors for DM, while other researchers generally only used one kind of method (LA, MA, etc.). Similar to other previous analyses, we also used national-level data in our analysis and identified new risk factors for DM. However, our data are not specifically designed for DM. Using the non-objective-designed data, we identified new risk factors for DM, while other studies commonly used objective-designed data. Meanwhile, we not only found new risk factors for DM but also re-confirmed some other risk factors for DM (Table 4), which were identified by previous research. Certainly, because of the data limitation, there are some factors that we could not re-confirmed in this analysis.

## Discussion and limitations

### Discussion

After using seven IML methods to analyze the anonymous census data of Japan, four new potential risk factors of DM were identified for the first time. In contrast, another 11 risk factors were reconfirmed using IML methods.

In contrast to Mika et al. [23] identifying that life environment affects DM, our analysis showed for the first time that insurance type and national pension would lighten the risk of DM in some aspects in Japan. Compared with stress, hypertension, and hyperlipidemia, the higher factor contribution of the national pension type shows us one possible direction to preventing DM: promoting one country’s pension system. Meanwhile, older citizens with insurance have fewer associated risk factors than those with other insurance (Table 2) in Japan. These certificates that Japan’s insurance system help prevent diabetes in some aspects. Japan has a unique insurance system and national pension system to protect citizens. Because of Japan’s unique healthcare system, the insurance type and national pension were identified as associated factors of DM in this study. However, the identification of insurance type and national public pension shows a possible governmental effort direction to prevent diabetes: offering an efficient healthcare system, which agrees with the opinion of Magriplis et al. [65] that immediate public health intervention is the primary prevention of type 2 diabetes.

Health awareness level was also identified as one possible risk factor associated with DM for the first time in this analysis. Our results indicate that people with good health awareness had fewer risk factors associated with DM (Table 2) than people with a general health awareness level. Meanwhile, the health awareness level has a comparatively higher correlation with stress than other factors, whose current reason is unclear. More profound research is necessary to understand the complex relationship among the various risk factors of DM.

While Chen et al. [11] found that age affected the prevalence of DM. This analysis found that the probability and the risk of DM increase with age, which matches our common sense that older people are easier to get various sicks. Meanwhile, like previous research [11, 12], which certifies that gender affects the prevalence of DM, factor gender also affected the risk of DM in our analysis. Our results show that males have a higher risk of DM (OR = 3) and more associated risk factors of DM (Table 2). The different risk between males and females tells us that males should be aware of the high possibility of having DM in Japan, and more efforts are needed to limit DM occurrence among males in Japan.

As one significant risk factor of DM, obesity was reconfirmed as a severe risk factor (OR > 6) in our study, which agrees with the finds of A. Brown et al. [21]. The higher risk of DM for obese Japanese people alarms us again that more effort is needed to halt obesity. At the same time, hypertension and hyperlipidemia don’t have highly adverse effects on DM as we imaged comparing with the factor insurance type (Table 3). The comparatively lower factor contribution of hypertension and hyperlipidemia with health awareness level tells us that more work should be focused on helping citizens to improve their health awareness level to prevent DM in Japan. Meanwhile, following Norito Kawakami et al.[42], we found that the professional category will affect the associated risk factors for DM (Table 2) in Japan, which clarifies that potential DM patients should be treated differently depending on their professional type.

Like previous research [12, 41, 43], our analysis also certifies that stress raises the risk of DM (OR > 1). To clarify the causes of stress in Japan, the statistical data on reasons for stress (Appendix 5) in Japan were checked. The top three reasons for stress in Japan were: disease and nursery, the problem of income balance, and problems with work. This tells us that the Japanese should be careful in easing the stress from ailments, income balance, and work, specifically for the stress from work, which was already certificated in previous research [12, 41, 43].

In contrast to other studies [10, 13, 25–38], which found that smoking status (or tobacco consumption) will affect the situation of type 2 DM, we found that smoking status did not have a high contribution to DM compared with risk factors such as obesity, age, and gender, and a newly identified factor: health awareness level. We also found that people, who smoked every day, had the same number of associated risk factors for DM as people who did not smoke in Japan (Table 2).

Separately from other studies, we found the number of family members as an associated risk factor for DM for the first time in Japan. However, the comparatively deeper relationship among factor number of family, room space (Table 3 and Fig. 2), and room number makes it difficult to explain the effects caused by the factor: family member. Future work needs to find how family structure can influence the risk of DM.

### Limitation

Certainly, limitations exist in our study. Firstly, the data used in this study are obtained from 2013 because Japan government hadn’t opened the newest data when we started this analysis. More recent data will be used in future investigations. Secondly, the data in this research does not classify the categories of DM because of the anonymous census data type. However, because of the non-objective-oriented census data, we can find new knowledge using interpretable machine learning methods. Finally, because of the complex situation and relation of risk factors in a realistic society, we should have analyzed the combined relationship among factors. Future studies should consider the compound relationship among risk factors. Despite the limitations, our findings point to a different aspect for identifying unknown risk factors of DM using non-objective-oriented designed data. Meanwhile, analysis using census data broadens the usage of big data, especially in today’s prosperous and intelligent society.

## Conclusion

No-objective-oriented census data were analyzed using various interpretable machine learning methods in this study, and 15 risk factors for DM were identified. Specifically, four new risk factors of DM: members of a family, insurance type, national pension type, and health awareness level were found for the first time in this analysis. Our study certifies that using interpretable machine learning methods can help us find new knowledge in our current big data society. Moreover, our analysis results provide a new direction to prevent diabetes in the current AI society. Certainly, our analysis clears some aspects of DM, and more risk factors of DM still exist. However, our study inspired research to find more risk factors associated with DM using non-objective-oriented data in the current data-prosperous society. Our study is also an efficient endeavor at data mining in the contemporary intelligent and big data society, which will widen the research border for future studies.

## Data Availability

Not applicable.

## Appendix 1

**Table 5:** The statistical data on stress reasons in Japan

**Reasons**	Number	**Reasons**	Number
1. Relationship of family	2550	12. Pregnancy, Delivery	168
2. Relationships except for family	2340	13. Childcare	452
3. Things about love and sex	377	14. Housework	906
4. Marriage	288	15. Study, Examination	565
5. Divorce	112	16. Children’s Education	1052
6. Bullying, Sexual Harassment, Harassment	118	17. Work	4790
7. Thins about life purpose	1798	18. Family work	1169
8. No free time	1254	19. Living and living environment (including pollution, safety, and traffic conditions)	1418
9. Income, household, debt, etc	4533	20. Other	1352
10. Self-illness and long-term care	5725	21. do not know the reasons	373
11. Family members’ illness and long-term care	2571	22. Unknown cause of worries and stress	1156

## Availability of data

The data used in this study need usage approval from the Ministry of Healthcare, Labor, and Welfare of Japan.
